# Targeting TGFβ with chimeric switch receptor and secreted trap to improve T cells anti-tumor activity

**DOI:** 10.3389/fimmu.2024.1460266

**Published:** 2024-10-24

**Authors:** Tatyana Matikhina, Cyrille J. Cohen

**Affiliations:** The Laboratory of Tumor Immunology and Immunotherapy, The Mina and Everard Goodman Faculty of Life Sciences, Bar-Ilan University, Ramat Gan, Israel

**Keywords:** T cells, cellular immunotherapy, chimeric cytokine receptor, TGF-β, TCR-T cells

## Abstract

**Introduction:**

TGFβ is a major immunoinhibitory factor present in the microenvironment of solid tumors. Various cancer types acquire the ability to overexpress TGFβ to escape immune response. Specifically, TGFβ dampens cytotoxic T cell activity, and its presence has been correlated with tumor invasion and poor prognosis.

**Methods:**

In this study, we developed two approaches to counteract the effects of TGFβ and provide a functional advantage to genetically engineered T cells in the immunoinhibitory tumor milieu. We designed a TGFβRI-based co-stimulatory switch receptor (CSRI), comprising the TGFβ receptor I extracellular binding domain and a 4-1BB co-stimulatory signaling moiety. Additionally, we tested the efficacy of a TGFβ-binding scFv trap produced by T cells.

**Results:**

We demonstrated that both approaches enhanced tumor-specific T cell cytokine secretion, upregulated activation markers, and reduced inhibition markers upon co-culture with melanoma targets. Furthermore, CSRI and the anti-TGFβ trap exhibited improved anti-tumor function *in vivo*.

**Conclusion:**

Overall, we show that targeting the TGFβ pathway can enhance cellular immunotherapy.

## Introduction

The transforming growth factor β (TGFβ) cytokine plays a crucial role in cell biology and is a critical regulator of the immune response. Overexpression of TGFβ has been detected in various cancers, including breast, colorectal, pancreatic, prostate lung cancers and melanoma (1). In the early stages of carcinogenesis, TGFβ exerts an inhibitory effect on the cell cycle. However, as the disease progresses, tumor cells acquire mechanisms to escape this inhibitory effect (2). In later stages, TGFβ even promotes tumor progression. This alteration in cancer cell response to TGFβ can result from changes in receptor expression or downstream signaling components. TGFβ influences the tumor microenvironment (TME), enhancing immunosuppression and favoring tumor dissemination (3). It can also promote the expression of pro-metastatic genes by tumor cells, such as matrix metalloproteinases (MMPs), which degrade the extracellular matrix and facilitate tumor cell migration. This can lead to the formation of distant metastases and decreased patient survival (4).

In the immune system context, TGFβ mediates the differentiation of T cells into T-regulatory cells and induces immunosuppression. TGFβ inhibits T cell proliferation and can influence their differentiation into pro- or anti-inflammatory cells depending on the additional cytokines present in the cellular milieu (5, 6). TGFβ induces differentiation of naïve CD4^+^ T cells into Foxp3^+^ T regulatory cells (Tregs) with suppressive functions against the expansion of antigen-specific T cells. Furthermore, it suppresses CD8^+^ T cell cytotoxic function. TGFβ interferes with the functions of dendritic cells (DCs) and cytotoxic natural killer (NK) cells, preventing tumor recognition by NK cells and impairing their cytotoxicity. It also skews the polarization of macrophages and neutrophils to a pro-tumorigenic phenotype (7). Blocking TGFβ signaling impairs tumor progression, enhances the antitumor response of CD8^+^ T cells, and increases the infiltration of NK cells and T cells to metastatic sites (6).

Active TGFβ binds to TGFβ Receptor I (TGFβRI) and TGFβ Receptor II (TGFβRII), forming a hetero-tetrameric complex consisting of two TGFβRI and two TGFβRII molecules (8). This complex formation activates the receptor kinases to phosphorylate downstream targets, namely the SMAD proteins. The active SMAD complexes then function as transcription factors, binding to DNA and affecting the expression of various genes. For instance, SMAD3 enhances Foxp3 expression, while SMAD2/3 inhibits granzyme B expression (9).

To further potentiate T cell-based cancer immunotherapy, recent strategies focus on blocking TGFβ activity. Some approaches demonstrating significant therapeutic efficacy in clinical trials include small molecule receptor kinase inhibitors and TGFβ-directed antibodies known as TGFβ traps (10). Other strategies involve expressing modified TGFβ receptors in T cells, such as truncated TGFβRII acting as a dominant negative receptor, or TGFβRII fused to a co-stimulatory molecule. The knock-in of chimeric TGFβRII, together with antigen-specific T cell receptors (TCRs), has enhanced anti-cancer T cell efficacy both *in vitro* and *in vivo* (11, 12).

Immunotherapy encompasses various cancer treatment approaches, including oncolytic virus therapy, cancer vaccines, cytokine therapies, immune checkpoint inhibitors, and adoptive cell transfer (ACT) (13). ACT uses tumor specific T-cells, either derived from tumor infiltrating lymphocytes (TILs) or genetically engineered T cells expanded *ex vivo* and has shown sustained clinical efficacy (14, 15). Currently, T cell specificity can be genetically engineered using two types of receptors, namely native TCRs and chimeric antigen receptors (CAR). Incorporating co-stimulatory molecules like CD28 or 4-1BB into 2^nd^ generation CARs has enhanced CAR T cell activity against cancer (15, 16). Additionally, one can supply T cells with co-stimulatory signaling using chimeric switch receptors (CSRs); for example, we have shown that T cells can be modified to derive benefit from the presence of inhibitory factors like TIGIT (17) or PD1 (18) ligands or sialic acids (19). CSRs can bind inhibitory ligands, whether membranal or soluble, via their extracellular (EC) domains but can transmit co-stimulatory signals through their intracellular (IC) domains. Such chimeric receptors have shown, for example, potent anti-tumor effects in the presence of inhibitory cytokines like IL4 (20). Administration of CSR-T cells with a PD1 ligand-binding domain and CD28 signaling moiety led to tumor regression in animal models and showed biological activity of modified T cells without adverse effects in patients (21–23).

In the present report, we aimed to create a TGFβRI-based CSR to improve T cell resistance to inhibitory TGFβ cytokine and potentially benefit from its presence in the Tumor Microenvironment (TME). This chimeric receptor, CSRI, included TGFβRI and 4-1BB co-stimulatory molecule. TGFβRI-based CSR significantly improved T cells activity *in vitro* and *in vivo* against cancer cells despite the presence of the inhibitory TGFβ cytokine. CSRI-equipped cells displayed lower levels of inhibitory receptors following long-term exposure to tumor cells. Additionally, we engineered T cells to produce and secrete a TGFβ blocker, which also mediated tumor growth delay *in vivo*.

## Materials and methods

### Donor PBMC and cancer cell lines

Peripheral Blood Mononuclear Cells (PBMCs) used in this study were obtained from healthy donors through the Israeli Blood Bank (Sheba Medical Center, Tel-Hashomer, Israel). The 938 melanoma cell line HLA-A2^-^/MART-1^+^ (938) (CVCL_8058) was generated at the Surgery Branch (National Cancer Institute, National Institutes of Health, Bethesda, MD) as previously described (24). The 938A2 line is an HLA-A2-transduced derivative of 938 using a pMSGV1 retroviral construct encoding the HLA-A*0201 molecule. SK-MEL23 is an HLA-A2^+^ melanoma cell line (25) (CVCL_6027). SK-MEL23 mCherry is a SK-MEL23 melanoma cell line transduced with mCherry. SK-MEL23 mCherry TGFβ is a SK-MEL23 mCherry cell line further transduced with the TGFβ1 encoding construct. A375 is an HLA-A2^+^/MART-1^-^ melanoma cell line (CVCL_0132). The viral packaging line 293GP (CVCL_E072), which stably expresses GAG and POL proteins, has been previously described (26), and was used for transfections. Adherent cells were cultured in Dulbecco’s Modified Eagle Medium (DMEM) (Invitrogen, Carlsbad, CA) supplemented with 10% heat-inactivated Fetal Bovine Serum (FBS) (Biological Industries, Beth Haemek, Israel) and maintained at 37°C in a 5% CO_2_ incubator. Lymphocytes were cultured in BioTarget medium (Biological Industries, Israel) supplemented with 10% heat-inactivated FBS and 300 IU/ml IL-2 and were maintained at 37°C in a 5% CO_2_ incubator.

### Construction of retroviral vectors

**Figure 1:**
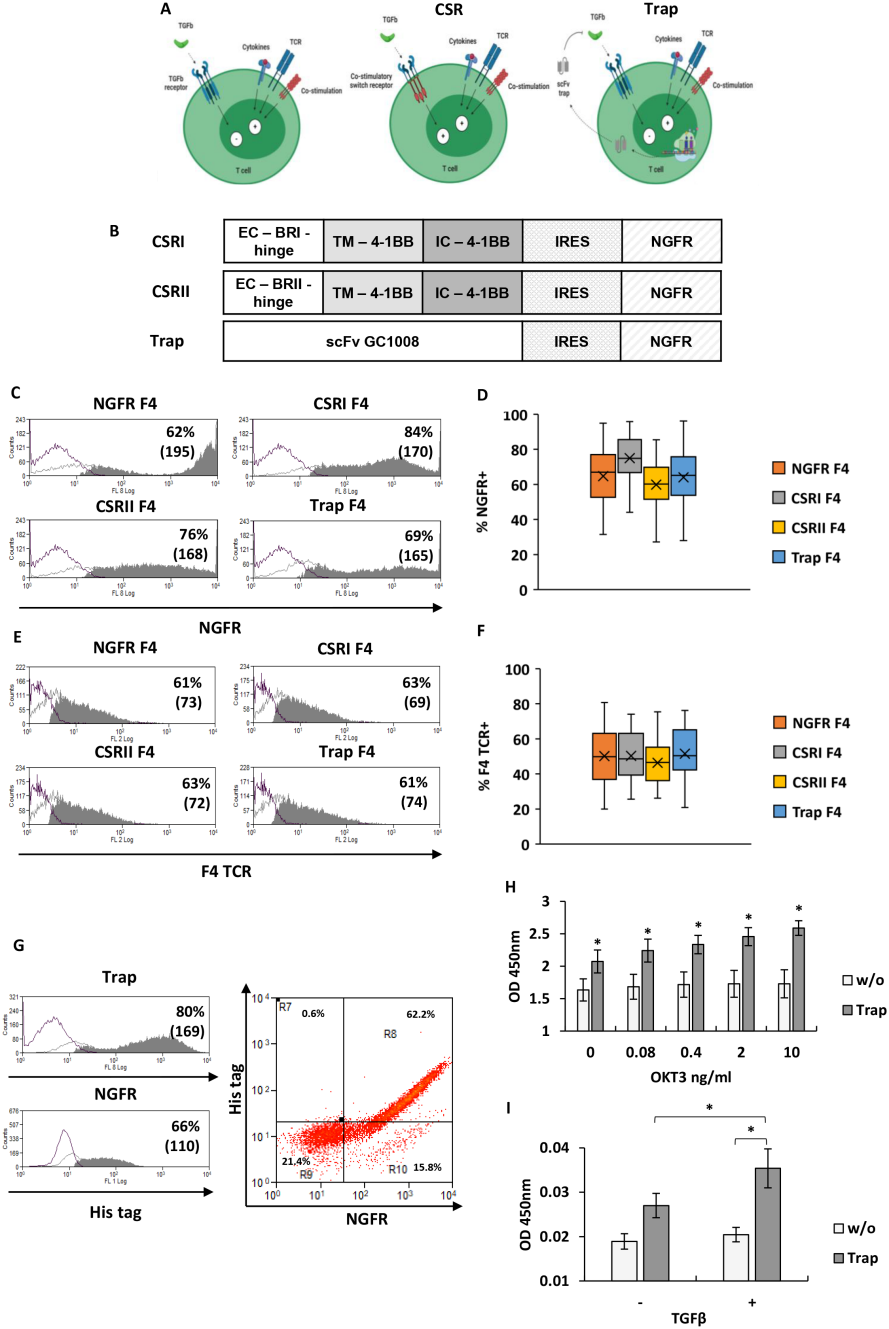
Design and expression of TGFβR-based CSRs and anti-TGFβ trap. **(A)** Schematic representation of the concept: central panel depicts the CSR approach, right panel the trap approach, compared to left panel which shows native state (generated with BioRender). **(B)** Schematic representation of CSRs and trap encoding constructs used in this study. (EC – extracellular domain, TM – transmembrane domain, IC – intracellular domain, IRES – Internal ribosome entry site) **(C)** T cells were transduced with retroviral constructs encoding the different CSR or trap constructs followed by an IRES-NGFR sequence, or the control gene only (NGFR). Cells were analyzed for transduction efficiency by flow cytometer following staining for NGFR expression. Representative histograms with the percentage of positive cells and mean fluorescent intensity (MFI - in bracket) are shown. **(D)** Boxplot summary of the transduction efficiency with the different constructs as indicated (n=15, with 15 different donors; bars represent SEM). **(E, F)** Similarly, following transduction with the F4 TCR, T cells were analyzed for Vβ12 expression: **(E)** representative histograms from one experiment and **(F)** a boxplot summary of the results of n=15 independent experiments (with 15 different donors). No significant difference in F4 TCR expression by T cells transduced with constructs was found (p=0.32, calculated using ANOVA test; n=15, with 15 different donors). **(G)** T cells transduced with the Trap-IRES-NGFR constructs were stained for NGFR expression and using an His-tag specific antibody to detect trap expression. Representative histograms of NGFR expression and of intracellular staining of His tag are shown on the left panel and a representative dot-plot of both staining is shown on the right panel. These results are representative of n=3 experiments with 3 different donors. **(H)** Trap secretion was evaluated following stimulation of transduced cells with plate bound OKT3 at the indicated concentrations ranging from 0 to 10 ng/ml. Trap secretion in the medium was measured by ELISA, using HRP-labeled anti-His tag. These results are presented as mean ± SEM of n=3 independent experiments with 3 different donors. The differences between trap and w/o (no transduction) were found statistically significant (*p<0.002, calculated using *Student’s* t-test). **(I)** Binding ability of trap to TGFβ. Supernatant collected from lymphocytes, transduced with trap or not (w/o), was incubated in plates previously coated with TGFβ (+) or not (-). ELISA was performed using a His-tag antibody to detect trap binding. The results are the mean ± SEM of n=4 independent experiments with 4 different donors. The difference between TGFβ trap and w/o control was found to be statistically significant, as the difference between TGFβ coated and non-coated plates (*p<0.05, calculated using a paired *Student’s* t test).

The retroviral backbone used in this study pMSGV1 has been previously described (27). The α- and β-chains from the previously characterized TCR specific for MART-1_26-35_ termed F4 (28), were subcloned into pMSGV1 (27). TGFβ CSRI and CSRII were generated by fusing the extracellular domain of TGFβR1 and TGFβR2 respectively to the transmembrane and intracellular region of 4-1BB (29) using overlapping PCR (**Figure 1**) (11). More specifically, these molecules were constructed using a mega-primer approach. The extracellular domains of TGFbR1 (aa 1-126) and TgfbR2 (aa 1-166) were amplified by PCR and fused to the hinge-intracellular region of 4-1BB (aa 175-255). The anti-TGFβ trap is a scFv antibody based on the variable heavy (V_H_) region and variable light (V_L_) regions of the anti-TGFβ antibody Fresolimumab (GC1008), linked by a (G_4_S)_3_ linker, followed by a 6xHis Tag and was synthesized (Genscript). These constructs were subcloned into pMSGV1. A truncated low-affinity Nerve Growth Factor Receptor (NGFR – aa 1-277) control gene was produced by PCR with a stop-codon following the transmembrane domain of the gene. To enable transduction efficiency tracking, NGFR under the control of an Internal Ribosomal Entry Site (IRES) was cloned following the CSRs or trap sequence (**Figure 1**). In addition, we cloned full-length mCherry and TGFβ1 into pMSGV1 for tumor cell transduction, using the following primers mCherry XhoI for:CGATCCTCGAGACCGCCATGGTGAGCAAGGGCGAGGAGG, mCherry EcoRI rev: TCTAGAGAATTCATTACTTGTACAGCTCGTCCATGCC, TGF beta1 Nco for: CATGCCATGGGGCCGCCCTCCGGGCTGCGGCTG and TGF beta1 Not rev: CATGGCGGCCGCTCAGCTGCACTTGCAGGAGCG.

### Determination of MART-1 transcript expression

To determine MART-1 transcript expression, total RNA was extracted from 2x10^6^ melanoma cells lines with Total RNA Mini Kit (Blood/Cultured Cell) (Geneaid, Taiwan) according to the manufacturer’s instructions. After digestion with RNAse-free DNAseI to eliminate the genomic contamination, the RNA was used to synthesize cDNA with AzuraQuant cDNA Synthesis Kit (Azura genomics, Raynham, MA, USA) according to manufacturer’s instructions. 50ng of cDNA was used as a template and subjected to PCR using the following primers: MART1 for: GTGTCACCATGGGGCCAAGAGAAGATGCTCACTTC and MART1 rev: CGATCAGCGGCCGCTTAAGGTGAATAAGGTGGTGGTGAC.

### Retroviral transduction

For transient virus production, 2.5 x 10^5^ 293GP cells were transfected with 2 μg of pMSGV1-subcloned retroviral construct DNA and 1 μg of envelope plasmid (VSV-G) using JetPrime transfection reagent, according to the manufacturer’s instructions (Polyplus, France). After 4 hours of incubation, the cell medium was replaced. Retroviral supernatant was collected 48 hours afterwards. Isolated Peripheral Blood Lymphocytes (PBLs) were stimulated with 50 ng/ml OKT3 (eBioscience, San Diego, CA) and plated at 2 x 10^6^ cells per 2ml in 24-well plates. The lymphocytes were cultured *in vitro* for 48 hours. Retroviral transduction was performed as follows (30): plates previously coated with RetroNectin (Takara, Otsu, Japan) and 2 ml of retroviral supernatant were centrifuged at 4°C and 2000g for 2 hours. After centrifugation, the supernatant was removed, and 1 ml of stimulated PBLs were added to each well at 0.5 x 10^6^ cells/ml (or 0.25 x 10^6^ cells/ml for cancer cells). Spinfection was performed by centrifuging the plates for 10 min at 1000g. Double transductions were performed, with the F4 TCR (on the first day) followed by the different constructs (2^nd^ day). After transduction, the cells were expanded at 37°C in a 5% CO_2_ incubator and split as necessary to maintain cell density between 0.5 and 3 x 10^6^ cells/ml.

### Flow cytometer analysis and antibodies

Anti-Vβ12 antibody specific for F4 TCRβ was purchased from Beckman-Coulter-Immunotech (Marseille, France). Fluorophore-labeled antibodies against human CD8, CD4, CD25 (IL2Rα), CD69, CD137 (4-1BB), CD134 (OX40), TIGIT, CCR7, CD45RO, TIM3, PD1, NGFR and HLA-A2 were purchased from BioLegend (San Diego, USA). The fluorophore-labeled anti-CD107a (lysosomal associated membrane protein 1) was supplied by Southern Biotechnology Associates (Birmingham, AL). Immunofluorescence was analyzed as the relative log fluorescence of gated live cells and the Mean Fluorescence Intensity (MFI), were measured using a CyAn-ADP flow cytometer (Beckman Coulter, Brea, CA, USA). Approximately 1 x 10^4^ cells, gated on live cells, were analyzed. Cells were stained in a flow cytometry staining (FACS) medium made of phosphate-buffered saline (PBS), 0.5% bovine serum albumin (BSA), and 0.02% sodium azide.

### Intracellular staining

For intracellular staining, 5 x 10^5^ cells were fixed with 5% formaldehyde and permeabilized using ice-cold 90% methanol for 20 minutes. The cells were then washed in FACS buffer, stained for His tag expression using a specific anti-His tag antibody (Miltenyi Biotec, Germany). Finally, the cells were analyzed using cytometry, gated on the lymphocyte population.

### ELISA and cytokine release assay

Peripheral blood lymphocytes (PBL) cultures were tested for reactivity in cytokine release assays using commercially available ELISA kits for IL-2, IFNγ and TNFα (R&D Systems, Minneapolis, MN, USA). For these assays, 1 x 10^5^ responder cells (T cells) and 1 x 10^5^ stimulator cells (tumor cells) were incubated in a 200 μl culture volume in individual wells of 96-wells plates. Human TGFβ1 (PeproTech, Rocky Hill, NJ, USA) was added to the co-culture volume at the final concentrations of 0.4 and 1.2 ng/ml where indicated. Stimulator cells and responder cells were co-cultured for 18 hours. Cytokine secretion was measured in culture supernatants, diluted to be in the linear range of the assay. Melanoma cancer cell lines were tested for TGFβ secretion using a commercially available ELISA kit for human/mouse TGFβ1 (Invitrogen, ThermoFisher Scientific, Massachusetts, USA), according to the manufacturer’s instructions. For this assay, 2 x 10^5^ cells of interest were incubated for 48 hours in a 200 μl culture volume in individual wells of 96-well plates. TGFβ secretion was measured in culture supernatants, diluted to fall within the linear range of the assay. Quantification of the cytokine was performed using a calibration curve. The results were obtained using an ELISA reader (ELx808 Biotek) at a wavelength of 450nm for the resulting color and 550nm to reduce the plastic background absorbance.

Trap secretion detection was performed using an ELISA. For this assay, 2 x 10^5^ of trap-transduced T cells were incubated for 48 hours in a 200 μl culture volume in individual wells of 96-well plates. Trap secretion was measured in culture supernatants. The ELISA microplate was pre-coated with Protein A at 1 μg/ml and incubated overnight. To further demonstrate TGFβ binding ability of the trap, the ELISA plate was precoated (or not) with TGFβ at 0.5 μg/ml and incubated overnight. After incubation the plate was washed with wash buffer (1x PBS with 0.05% tween) and blocked with 1% Bovine Serum Albumin (BSA) solution for 1 hour. Then, the sample was applied and incubated for 2 hours. Following washing to remove unbound antigen, HRP-conjugated anti-His tag antibody (Biolegend) was added (0.5 μg/ml) for 2 hours. TMB was added to the plate, and the colorimetric reaction was stopped by the addition of 1M H_2_SO_4_. The results were obtained using ELISA reader (ELx808 Biotek) at a wavelength of 450 nm for the resulting color and 550 nm to reduce the plastic background absorbance.

### Cell mediated cytotoxicity assay

SK-MEL23 mCherry TGFβ target cells were co-cultured with effector transduced lymphocytes in 96-well tissue culture plate at Effector: Target ratios of 1:1, 2:1, 3:1, 5:1 respectively. All the wells were replenished up to a final volume of 200 μl of cells medium and incubated at 37°C for 24 hours. The co-culture wells were imaged every 2 hours, and the total orange integrated intensity (OCU x µm²/Image) was measured (and normalized to t=0) using the Incucyte system for image acquisition and live-cell analysis (Sartorius, Germany).

### *In vitro* hypofunction induction assay

T cells hypofunction was induced by repetitive antigen exposure. A total of 1 x 10^6^ transduced lymphocytes were co-cultured with 1 x 10^5^ engineered SK-MEL23 mCherry TGFβ target cells. Every 2 days, the same effector cells were transferred to a new culture vessel containing 1 x 10^5^ fresh tumor cells. This process was repeated three times over a total of 8 days. At the end of the 8-day co-culture, T cells were stained for markers related to T cell exhaustion and analyzed by flow cytometry.

### *In vivo* cytotoxicity assay

Twelve-week-old NOD/SCID/Gamma mice (Harlan, Jerusalem, Israel) were subcutaneously inoculated in the flank with a mixture of 2 x 10^6^ SK-MEL23 mCherry TGFβ cells and 2 x 10^6^ transduced lymphocytes resuspended in 100 μl Hank’s Balanced Salt Solution (HBSS) medium (Biological Industries, Beth Haemek, Israel) and 100 μl Cultrex matrix (Trevigen). Tumor size was measured every 2-3 days in a blinded manner using a caliper. Tumor size was calculated using the formula: (D x d^2^) x π/6, where D is the largest tumor diameter and d is the perpendicular one. Criteria for anti-tumor efficacy was considered a delay in tumor development in treated mice, compared to NGFR positive control. Animals were humanely euthanized if the tumor exceeded 1500 mm^3^. This study was carried out in accordance with the recommendations of the Bar-Ilan University Committee for Animal Welfare and the Israel Ministry of Health. The protocol was approved by the Bar-Ilan University Committee for Animal Welfare.

### Statistical analysis

The results presented in this study are expressed as the mean ± SE (SEM) of several assays with at least three different donors. Data were subjected to statistical evaluation using *Student’s* t-test or ANOVA test. Tumor sizes were compared using *Student’s* t-test or mixed model Anova. Mice survival data were plotted as a Kaplan-Meyer curve and compared using the LogRank test. p ≤ 0.05 was considered statistically significant.

## Results

### TGFβR-based CSRs or anti-TGFβ trap design and their expression

The TGFβ receptor (TGFβR) is a heterotetrameric receptor consisting of two TGFβRI and two TGFβRII subunits, which typically conveys inhibitory signals in effector T-cells (8, 9). Our goal was to engineer T cells with TGFβ-specific CSRs that, upon TGFβ binding, transmit a co-stimulatory signal instead of an inhibitory one (**Figure 1A**). Specifically, we constructed CSR variants based on TGFβRI or TGFβRII. TGFβRI-based chimera was named CSRI, while the TGFβRII-based chimera used as a positive control, was designated CSRII (11). We also evaluated an alternative approach to target TGFβ by engineering T cells to secrete a TGFβ blocking antibody acting as a “trap”. This TGFβ trap was based on a single-chain variable fragment (scFv) derived from Fresolimumab (GC1008 antibody). These constructs were cloned into a retroviral vector, followed by an IRES-NGFR sequence to enable the assessment of transduction levels (**Figure 1B**).

To endow T cells with tumor specificity, we transduced them with a MART-1 specific TCR (F4) previously used in clinical trials (31). Primary human T cells underwent a double transduction process - first with the F4 TCR (to ensure uniform TCR expression across the different groups) and then, either the CSR chimera, trap or NGFR (control) constructs. T cells were successfully transduced with the different constructs, reaching between 60-80% expression levels (**Figures 1C, D**). Similarly, the proportion of F4 TCR positive T cells was 61-63% (**Figures 1E, F**).

We further characterize the expression of the TGFβ-trap by T cells. To do so, we co-stained T cells transduced with the trap construct using anti-NGFR and intracellularly with an anti-His antibody. Flow cytometry analysis revealed a co-staining pattern in 62% of the cells (**Figure 1G**). Moreover, we tracked TGFβ-trap secretion by transduced cells. Following activation (or no activation), culture supernatant was incubated in ELISA plate to allow the binding of the secreted trap. We found a significant correlation between T cells stimulation and trap secretion, as indicated by the Pearson correlation test (r=0.805, p=0.05). Increased T cell stimulation resulted in a significant rise in trap secretion compared to both non-transduced w/o control cells and less stimulated T cells (**Figure 1H**). Finally, the anti-TGFβ trap was tested for its TGFβ-binding ability using an ELISA. For this, microplates were pre-coated with TGFβ at a concentration of 0.5 μg/ml. Supernatant from trap-transduced T cells was then applied to these plates to detect the presence of the trap (using anti-His). Significantly higher amounts of trap were detected in the TGFβ-pre-coated wells compared to non-precoated wells (p=0.03), (**Figure 1I**) indicating the capacity of this antibody to bind TGFβ.

### TGFβ secretion by cancer cell lines

**Figure 2:**
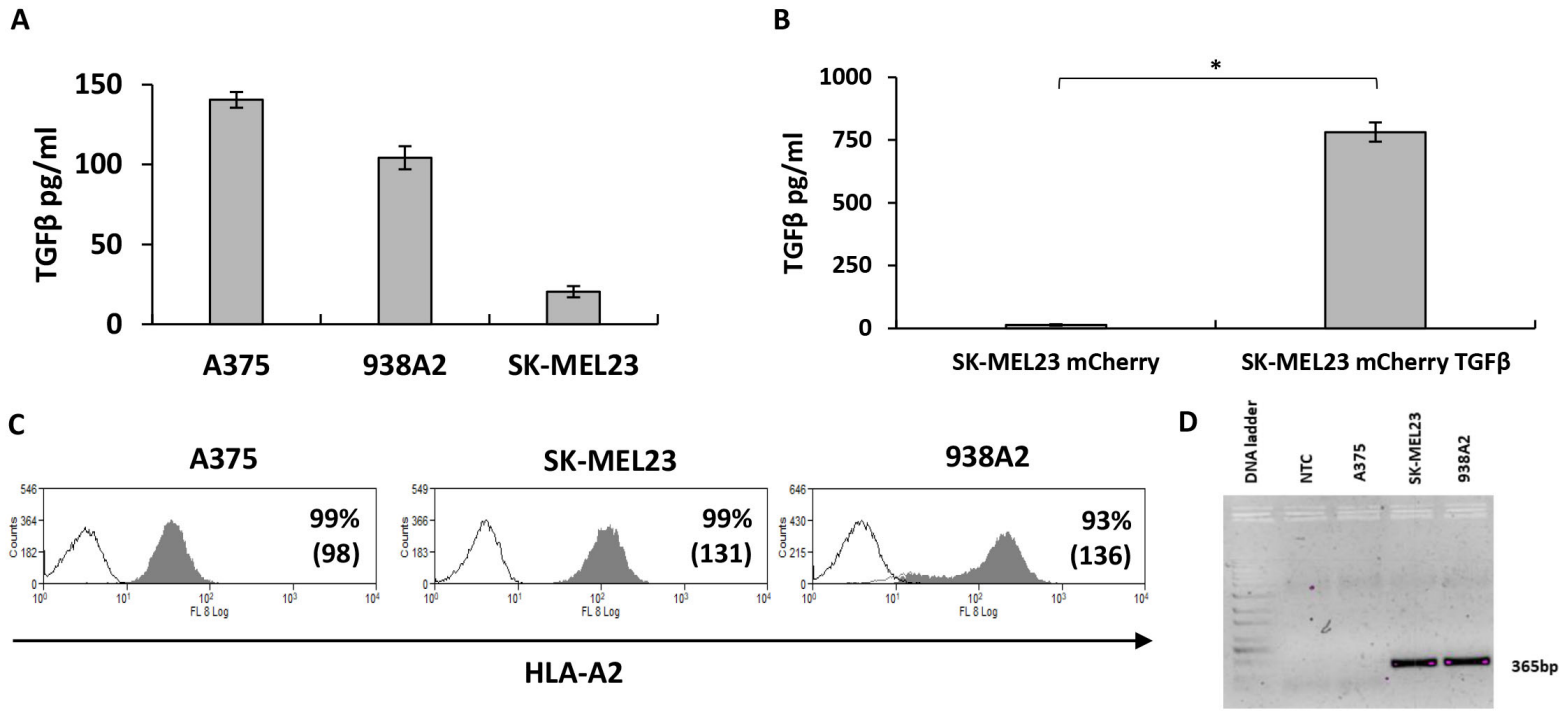
TGFβ and MART-1 antigen expression by melanoma cells lines. **(A)** Culture medium from melanoma cell lines as indicated was collected to determine TGFβ concentration using ELISA. The results are presented as mean ± SEM of n=3 independent experiments, the means difference found to be significantly different (p=0.07*10^-9^, calculated using ANOVA). **(B)** SK-MEL23 cell line was engineered to express mCherry, followed by transduction with TGFβ sequence-bearing vector to enhance TGFβ secretion. Supernatant from SK-MEL23 mCherry and SK-MEL23 mCherry/TGFβ cultures was evaluated for TGFβ content using ELISA. The results are presented as mean + SEM and the difference between the parental and engineered cell line was found to be statistically significant (*p=3*10^-5^, calculated using *Student’s* t-test). **(C)** Melanoma cells lines were analyzed using flow cytometry for HLA-A2 expression, essential for F4 TCR-dependent T cells activation. Representative histograms show the percentage of positive cells and mean fluorescent intensity (MFI in bracket). **(D)** For MART-1 antigen expression detection, total RNA was extracted from melanoma cell lines, followed by mRNA conversion to cDNA. The antigen expression was determined using PCR amplification with primers specific to a 365bp part of a gene, where cDNA was used as a template (NTC - No Template Control).

TGFβ has been shown to be expressed by tumor cells. Thus, we examined TGFβ secretion levels by the cancer cell lines we used in this study. As shown in **Figure 2A**, different melanoma cell lines secreted varying amounts of TGFβ – for example, 938A2 cells secreted over 100 pg/ml of TGFβ after 48 hours incubation. We also engineered the SK-MEL23 cell line to express TGFβ reaching concentrations of approximately 780 pg/ml (**Figure 2B**). We also characterized the A375, SK-MEL23, and 938A2 cell lines for HLA-A2 expression, which is necessary for the presentation of the MART1 epitope recognized by the F4 TCR (**Figure 2C**), reaching over 93% expression. As expected, we confirmed the expression of MART-1 antigen transcript by PCR in the SK-MEL23 and 938A2 cell lines but not in the A375, which served as an antigen-negative control in this study (**Figure 2D**).

### TGFβRI-based CSR and trap enhance T cells function and pro-inflammatory cytokines secretion

After evaluating transduction efficiency, we tested the functional capacity of T cells to enhance TCR-driven anti-cancer response. Constructs transduced T cells, along with F4 TCR, were co-cultured with melanoma cell lines at a 1:1 effector-to-target ratio, with the addition of soluble TGFβ. Following the co-culture, we measured the levels of Tumor Necrosis Factor-α (TNFα), Interferon-γ (IFNγ) (32) and Interleukin-2 (IL2), important for T-cell anti-tumor activity and proliferation (33).

**Figure 3:**
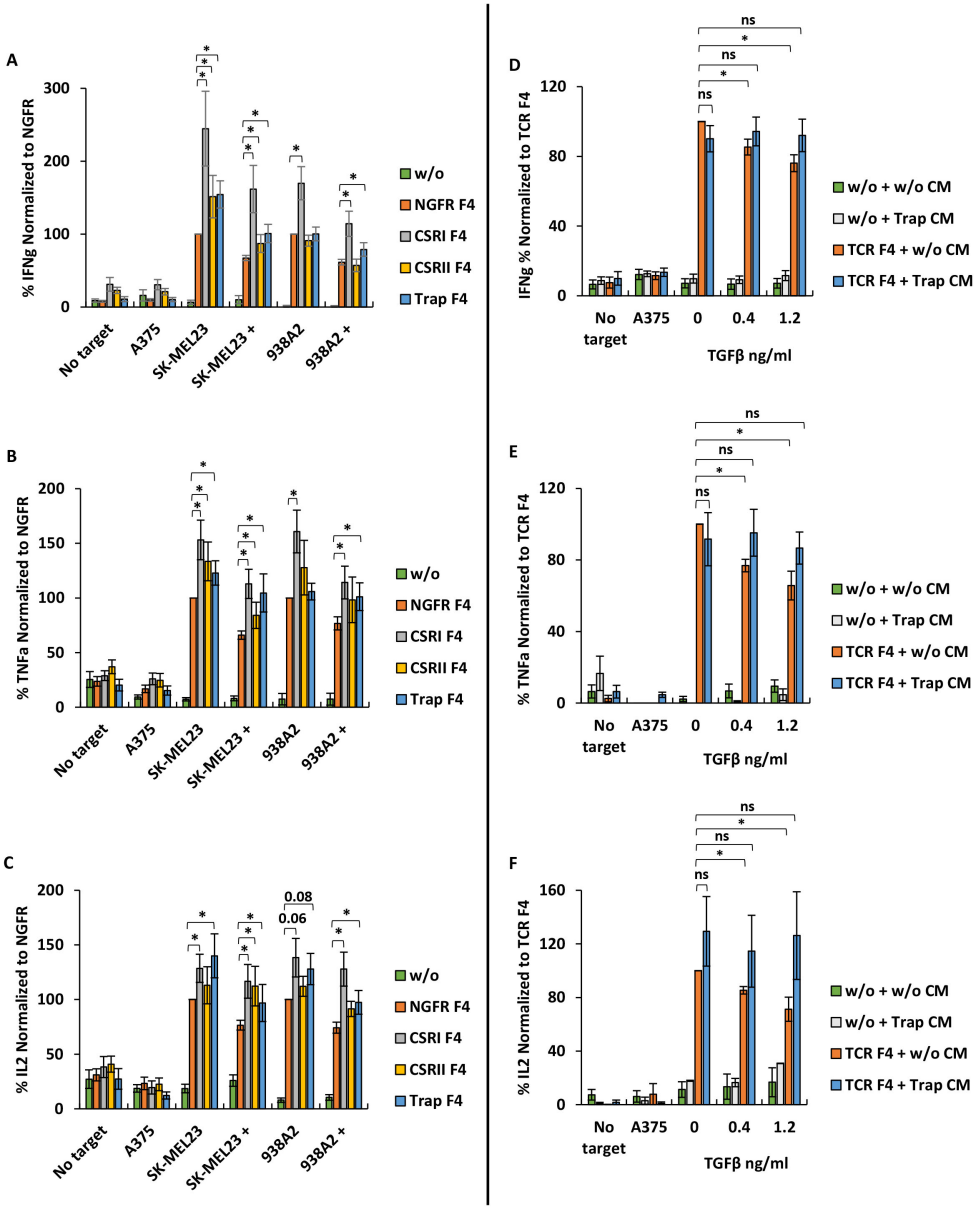
CSRI and anti-TGFβ trap enhance T cells pro-inflammatory cytokine secretion. **(A–C)** Human primary lymphocytes were transduced to express the F4 TCR along with CSRs, trap or NGFR only (control); w/o represents mock transduced lymphocytes. Transduced cells were co-cultured with melanoma cell lines SK-MEL23, 938A2 and A375 (control) along with TGFβ (1.2 ng/ml) or not. IFNγ **(A)**, TNFα **(B)** and IL2 **(C)** secreted to the co-culture media was measured by ELISA. The results are presented as mean ± SEM, normalized to NGFR F4 (control) (n=7 with 7 different lymphocytes donors, *p ≤ 0.05, calculated using a paired *Student’s* t test). **(D)** Human primary lymphocytes transduced with trap were cultured for 48 h and their medium (CM – conditioned medium) was isolated to evaluate the function of the secreted trap. In parallel, human primary lymphocytes were transduced to express F4 TCR or not (w/o – without, represents mock-transduced control). Following transduction, T cells were co-cultured with melanoma cell line SK-MEL23 and A375 (control) along with TGFβ (0.4 -1.2 ng/ml) or not (0 ng/ml) in the trap containing medium (CM) isolated as aforementioned. Following the co-culture, IFNγ **(D)**, TNFα **(E)** and IL2 **(F)** secretion in the co-culture was measured using ELISA. The results were normalized to F4 TCR-transduced T cells co-cultured in CM of mock-transduced T cells without TGFβ. The results are presented as mean ± SEM (n=4 with 4 different donors, *p<0.05, n.s., not significant, calculated using a paired *Student’s* t test).

As shown in **Figure 3A**, we noted that CSRI significantly enhanced IFNγ secretion by engineered T cells compared not only to NGFR F4 control (increasing secretion by 52-145%, p<0.006), but also to the other constructs (increasing IFNγ secretion by 35-94%, p ≤ 0.007). TGFβRI-based CSR contributed more efficiently to T cell cytokine secretion than TGFβRII-based CSR (20-51%, p ≤ 0.03) in co-culture against SK-MEL23 or the anti-TGFβ trap (17-54%, p<0.03). The anti-TGFβ trap was designed to counter the inhibitory effect of TGFβ by reducing the amount of free TGFβ in the co-culture volume. Indeed, Trap F4 T cells were more effective in mediating IFNγ secretion compared to the NGFR F4 control. We also observed that the addition of TGFβ induced a significant decrease in IFNγ secretion by NGFR F4 control cells of approximately 33-38% (p ≤ 9x10^-10^). While CSRs or trap constructs could not fully eliminate the inhibitory effect of TGFβ, they significantly mitigated it, and improved T cells function. Similar results were observed for TNFα and IL2 secretion (**Figures 3B, C**). No significant cytokine secretion was observed in co-culture with the negative control cell line A375.

To further characterize the activity of the TGFβ trap construct, we collected conditioned medium (CM) from cultures of T cells transduced with trap or mock-transduced. We set up a co-culture of F4-transduced T cells in the presence of trap CM or not. Additionally, TGFβ was added to the co-culture at concentrations of 0.4 and 1.2 ng/ml. As expected, we observed a decrease of nearly 25% (p<0.02) in IFNγ secretion in the presence of TGFβ and CM from mock-transduced cultures. However, this decrease was abrogated in the presence of CM derived from trap-transduced T cells (**Figure 3D**). A similar pattern was observed when measuring the secretion of TNFα and IL2 (**Figures 3E, F**).

These results suggest that CSRI can effectively enhance T cells anti-cancer pro-inflammatory function in the presence of TGFβ immuno-inhibitory cytokine. Additionally, the secreted trap can neutralize TGFβ, thereby contributing to preserving T cell function.

### Phenotypic characterization of engineered T cells

We next aimed to determine if the different constructs could influence the expression of activation markers on T cell surface. Upon activation, T cells can upregulate different activation markers such as CD69 (an early activation marker), members of the TNF receptor superfamily including co-stimulatory molecules like CD134 (OX40) and CD137 (4-1BB) or CD25 (IL2 Receptor α-chain) (34). TGFβ can influence T cell activation status, as it can downregulate marker expression such as in the case of CD25 (35). To assess the impact of engineering T cells with CSR/trap constructs, we co-cultured T cells expressing the latter and F4 TCR with melanoma cells in the presence of 1.2 ng/ml TGFβ.

**Figure 4:**
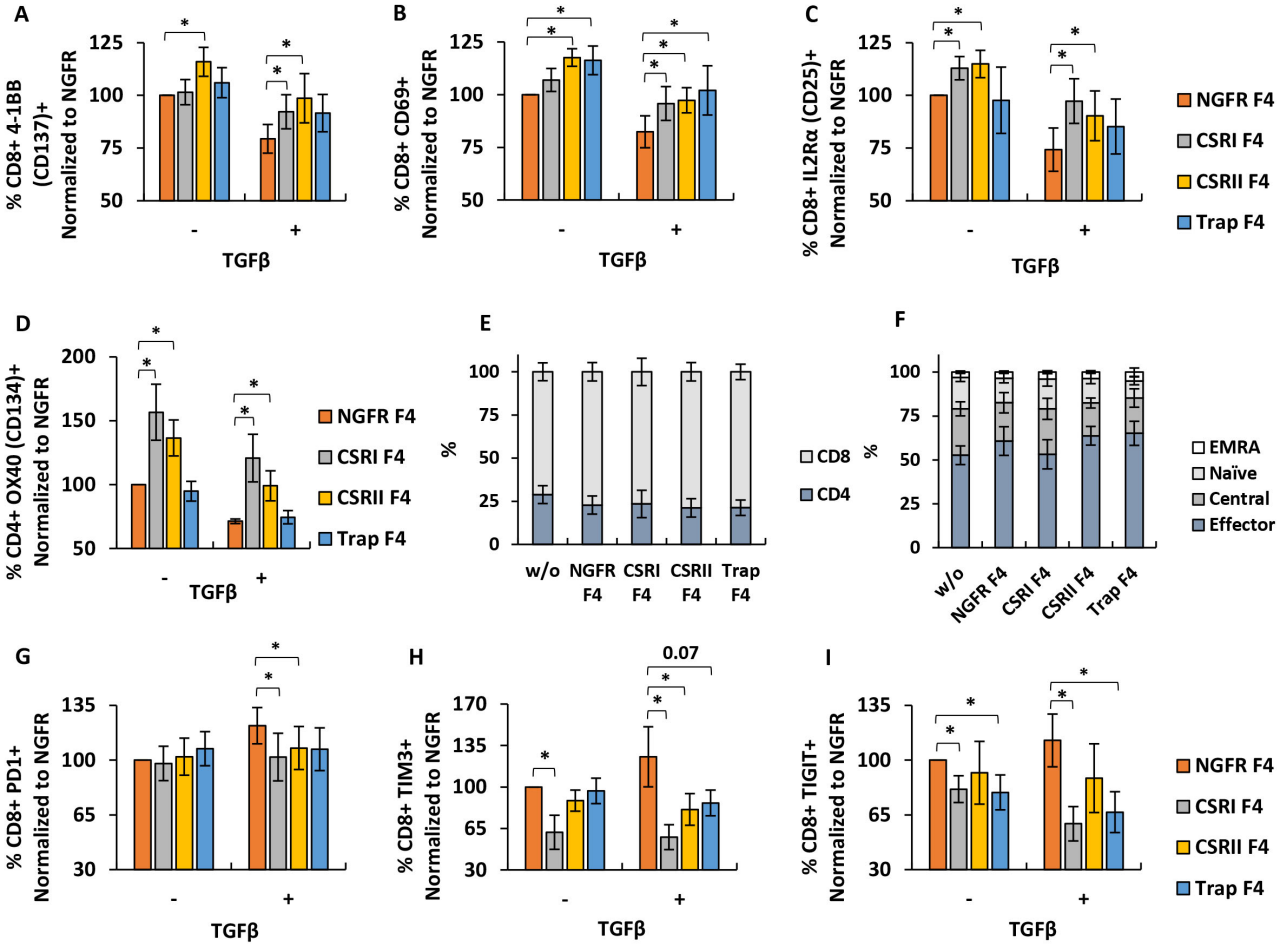
Phenotypic characterization of engineered T cells. **(A–D)** T cells were transduced with F4 TCR and CSRs, trap or NGFR only (control) and then co-cultured with SK-MEL23 with 1.2ng/ml TGFβ (+) or without (-). After the incubation, co-cultured lymphocytes were stained and analyzed by FACS, to detect the expression of 4-1BB **(A)**, CD69 **(B)** and IL2Rα **(C)** gated on the CD8^+^ population and of OX40 **(D)**, gated on the CD4^+^ population. These results were normalized to the expression observed in the NGFR F4 (control) population, co-cultured with SK-MEL23 without additional TGFβ (-). Results are presented as mean ± SEM (n=4 with 4 different donors, *p<0.05 calculated using a paired *Student’s* t test). **(E)** Following transduction, T cells were stained to determine CD4^+^/CD8^+^ distribution by flow cytometry. The results are presented as mean ± SEM of n=6, with 6 different donors and no significant difference was detected (p>0.1, calculated using *Student’s* t-test). **(F)** Memory phenotype was also assessed by staining with CD45RO and CCR7, followed flow cytometry to detect the percentage of naïve, central memory (TCM), effector memory (TEM) and terminally differentiated effector memory (TEMRA) T cells in the transduced lymphocytes population. The results are presented as mean ± SEM of n=5, with 5 different donors. No statistically significant difference was detected (p>0.1, calculated using *Student’s* t-test). **(G–I)** T cells transduced with F4 TCR and CSRs, trap or NGFR (Control) were repeatedly co-cultured at an E:T ratio of 10:1 with parental melanoma line SK-MEL23 mCherry (-) or its TGFβ-transduced version (+). Every 2 days for a total of 8 days, fresh tumor targets were added to the co-cultures (total of 4 instances). At day 8, T cells were stained for the expression PD1 **(G)**, TIM3 **(H)** or TIGIT **(I)**, gated on the CD8^+^ population and analyzed by flow cytometer. Results are presented as mean ± SEM and normalized to that of NGFR F4 co-cultured with SK-MEL23 mcherry (-) (for **(G)** n=11, with 11 different donors and for **(H, I)**, n=6. with 6 different donors and *p<0.05, calculated using a paired *Student’s* t test).

As expected, we observed that TGFβ significantly decreased activation markers expression in NGFR F4 control T cells (reduction by 20-30%, p<0.05). Although CSRs F4 T cells showed reduced activation markers expression due to the presence of TGFβ, this was more pronounced than in the NGFR F4 control. CSRI F4-transduced T-cells expressed more 4-1BB (increase of 13%, p=0.05), CD69 (increase of 14%, p=0.03), CD25 (increase of 23%, p=0.003) and OX-40 (increase of 50%, p=0.04) compared to NGFR F4 in the presence of TGFβ. Similar results were observed in T cells expressing CSRII. For Trap F4 T cells, we observed a significant improvement in CD69 expression (20%, p=0.02) despite the presence of TGFβ compared to NGFR F4. We noted that compared to CSRs, the TGFβ trap did not significantly influence the upregulation of activation markers, which could be due to a lack of co-stimulation signaling compared to that mediated by the chimeric receptors (**Figures 4A–D**).

Additionally, we characterized the ratio of CD4^+^ and CD8^+^ in the transduced populations and did not note a difference in CD4^+^/CD8^+^ phenotype distribution compared to NGFR control (p>0.1) (**Figure 4E**). We further assessed the memory phenotype of these populations. Transduced T cells were stained for CD45RO and CCR7 expression. Memory state analysis revealed that most of the cells in the T cells population displayed an effector memory phenotype (about 60% of the cells), with fewer exhibiting central memory characteristics (about 22%), followed by naïve (about 15%), and the smallest group being EMRA cells (about 3%). No significant differences in memory phenotype were detected in T cells transduced with the different constructs compared to NGFR control cells (p>0.1) (**Figure 4F**).

Since transduced T cells showed improvement in pro-inflammatory cytokines secretion and activation state compared to NGFR F4 control, we aimed to determine if these constructs can provide an anti-TGFβ protective effect on T cells after repeated exposure to antigen and under immune-inhibitory settings. We performed 3 consecutive co-cultures at a 10:1 E:T ratio, with transduced lymphocytes, replenishing every 48 hours the target cells (SK-MEL23 mCherry or SK-MEL23 mCherry TGFβ). After these 3 cycles, T cells were stained for inhibition markers expression, namely TIM3, PD1 and TIGIT. As seen in **Figures 4G–I**, CSRI provided significant protection against T cells exhaustion despite TGFβ inhibitory effect, as measured by the reduced expression of TIM3 (reduction of 68%, p=0.04), TIGIT (reduction of 54%, p=0.03) and PD1 (reduction of 20%, p=0.02) compared to NGFR F4 control cells. CSRII F4- transduced T cells showed a slightly less sensible reduction in checkpoint expression. For Trap F4 T cells, the effect was less pronounced however, we did note reduced levels of TIGIT (reduction of 46%, p=0.04) compared to NGFR F4. Thus, CSRs and trap constructs facilitate an increase in the expression of activation markers and a reduction in checkpoint expression in the presence of the inhibitory cytokine TGFβ.

### T-cells transduced with CSRs and trap mediate *in vivo* anti-tumor activity

**Figure 5:**
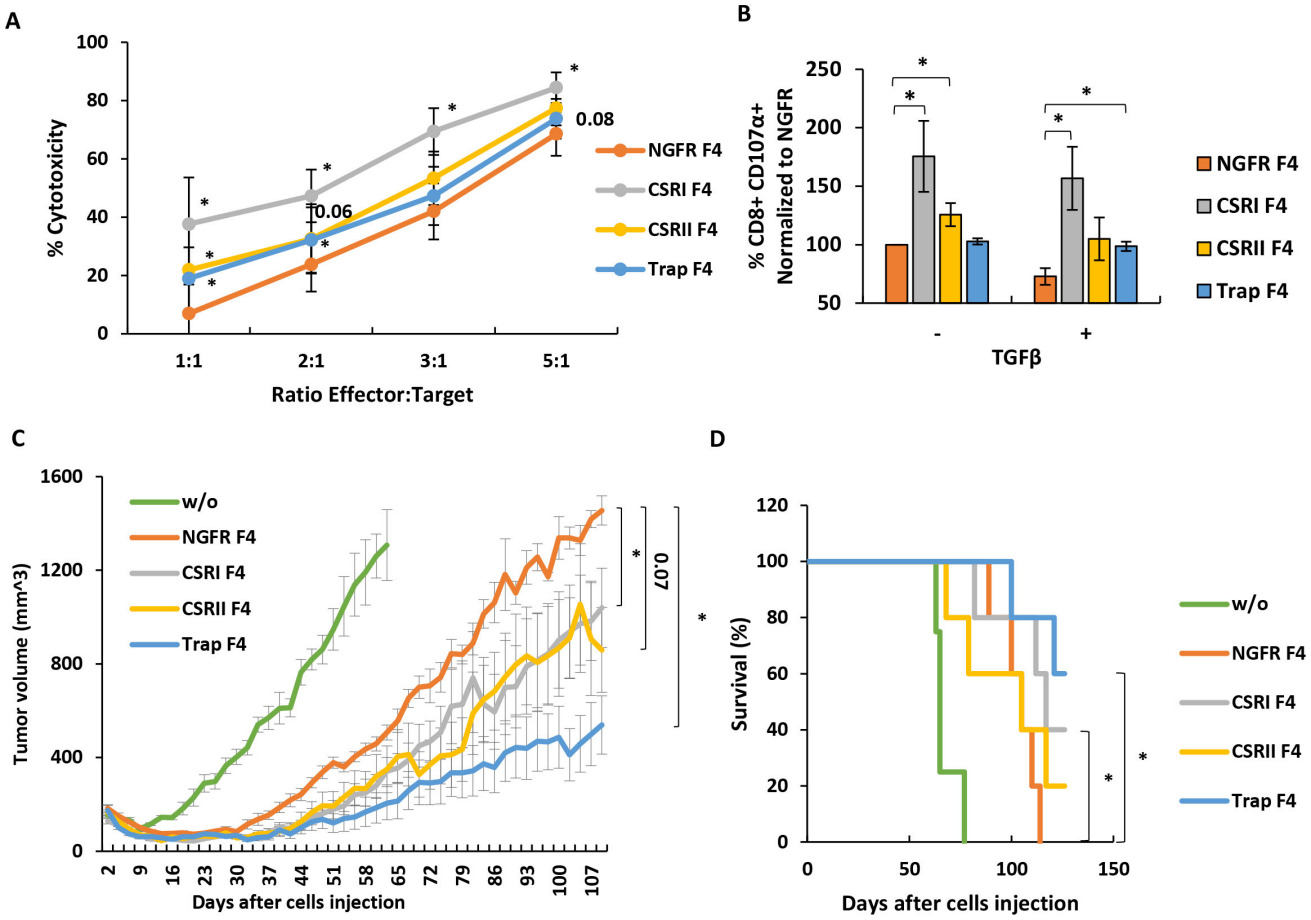
CSRI and anti-TGFβ trap can contribute to anti-tumor cytotoxicity *in vitro* and in a xenograft model. **(A)** Transduced T cells, expressing F4 TCR and CSR, trap or NGFR (control) were co-cultured with TGFβ-transduced SK-MEL23 mCherry target cells. Co-culture was performed at different E:T ratios as indicated. Fluorescent signal (OCU x µm²/Image) of live tumor cells (and normalized to t=0) was measured by Incucyte, every 2 h for 24h. Cytotoxicity was calculated as 100% - live cells %. Results at different E:T ratios are presented as mean ± SEM, n=6, with 6 different donors, and *p ≤ 0.05, calculated using a paired *Student’s* t test. **(B)** Additionally, these T cells were analyzed by flow cytometry for expression of CD107 degranulation marker after a short co-culture (2 h) with SK-MEL23 tumors targets in the presence (+) or not (-) of TGFβ. The results were normalized to that of NGFR F4 (control) co-cultured with SK-MEL23 without additional TGFβ (-) and are presented as mean ± SEM (n=5, with 5 different donors, *p<0.05 calculated using a paired *Student’s* t test). **(C)** T cells expressing F4 TCR and CSR, trap or NGFR were evaluated for their *in vivo* function. Briefly, NOD/SCID/Gamma mice were inoculated with 2 x 10^6^ TGFβ-transduced SK-MEL23 cells along with 2 x 10^6^ engineered lymphocytes in Cultrex. Tumor size (mm^3^) was measured using a caliper and tumor size calculated using following formula: D x d^2^ x π/6, where D is the largest tumor diameter and d is a perpendicular one. Results are presented as mean ± SEM (n=5, *p<0.05, calculated using *Student’s* t-test). The criteria for anti-tumor efficacy were based upon a significant delay in tumor development in treated mice, compared to NGFR positive control. **(D)** Mice survival was also evaluated and presented as a Kaplan-Meier plot, (with *p<0.05, calculated using LogRank test).

Next, we tested the influence of the different constructs on cytotoxicity. For this purpose, we co-cultured transduced T cells with mCherry-labelled SK-MEL23 TGFβ cells at different E:T ratios for 24 hours. In most cases, T cells engineered with either CSR or traps displayed significant cytotoxicity capacity compared to the NGFR F4 control. For example, at 1:1 E:T ratio we measured a proportion of 38%, 22% and 19% dead cells for CSRI, CSRII and Trap F4 T cells compared to 7% for the NGFR F4 control (**Figure 5A**, p<0.05). We also measured CD107 degranulation marker expression on T cells after co-culture experiments as another cytotoxicity parameter. As shown in **Figure 5B**, CSR expressing cells demonstrated an increase in CD107 expression (up to 75% for CSRI F4 compared to NGFR F4 control). While this effect was mitigated in the presence of TGFβ, we observed higher CD107 expression levels in T cells expressing the different constructs. For example, we observed a 27% decrease in CD107 expression for NGFR-F4 cells compared to conditions in which we did not add TGFβ (p=0.02). Trap F4 T cells were insensitive to the addition of TGFβ (**Figure 5B**).

Finally, we examined the anti-tumor function of T cells transduced with F4 TCR, and either CSRs or trap in an *in vivo* tumor xenograft model. 2 x 10^6^ SK-MEL23 TGFβ cells, along with 2 x 10^6^ transduced lymphocytes, were injected into the flanks of immunodeficient mice. The tumor size was measured every 2-3 days. As seen in **Figure 5C**, CSRI F4 transduced T cells contributed to a significant delay in tumor progression compared to NGFR F4 control cells (p=0.04). Interestingly, Trap F4 T cells displayed a better control of tumor progression. This was evident when analyzing survival curves (**Figure 5D**) with 60% of the mice still alive at the experiment endpoint for the trap group compared to none for the control group (p=0.01). In conclusion, Trap F4 transduced T cells mediated the most significant *in vivo* activity.

## Discussion

TGFβ can stimulate tumor progression and concomitantly inhibit the anti-cancer immune response. As it is a prominent component of the tumor microenvironment, we aimed to develop different approaches to counter TGFβ effects in genetically engineered T cells.

One approach we adopted was to express a chimeric switch receptor, that upon TGFβ binding, provides a co-stimulatory signal to the T cell. This confirms and extends a previous report that demonstrated a TGFβRII/4-1BB chimeric receptor mediated increased T cell function *in vivo* (11). Here, we observed that TGFβRI – based receptors demonstrated a similar activity adding to the versatility of the approach and suggesting that this receptor is amenable to molecular changes. One may suggest that combinations of co-stimulatory molecules are not limited to CD28 or 4-1BB and such TGFβRI CSR may also incorporate signaling moieties derived from additional stimulatory molecules such as OX40, CD27, ICOS or IL7R, for example (15, 36). Herein, we focused on a TCR model specific for melanoma. Although chimeric antigen receptors incorporate built-in co-stimulation, it is conceivable that CAR T cells may also benefit from this approach (36).

As aforementioned, we compared the activity of chimeric switch receptor based on TGFβRI (CSRI) or TGFβRII (CSRII). Both, anti-TGFβ CSRs showed effectiveness in various functional assays, but CSRI was more effective in pro-inflammatory cytokines secretion than CSRII. Although these receptors could not fully eliminate the inhibitory effects induced by TGFβ, CSRI-transduced T cells displayed a better functional profile. Both CSRI and CSRII contributed to improved functionality of T cells in cytotoxicity tests and demonstrated an ability to delay a tumor progression. The exact mechanisms behind the observed differences in effectiveness between CSRI and CSRII remain unclear and warrant further investigation. While the affinity of TGFβRII to its ligand is considered higher (8), it is possible that specific domains derived from TGFβRI confer higher affinity for TGFβ in the context of the chimeric receptor. It is also possible that since we replaced TGFβRI inhibitory domain by a costimulatory one in CSRI, we were able to reach a higher activation state than the other way around (CSRII and native TGFβRI), as the native TGFβRII does not naturally bear a signaling moiety and may not interfere with CSRI. Additionally, factors such as receptor expression levels, localization, or downstream signaling may also influence the overall functionality of the chimeras.

Besides making use of CSR, we explored an additional approach to counter the effects of TGFβ by preventing its binding to TGFβR using a secreted antibody-based trap. A potential advantage of this approach over CSRs lies in the ability to widely target TGFβ in the TME, thereby reducing its effects systematically - this could influence also non-engineered cells present in the vicinity of trap secreting TCR T cells. This blocking activity can even occur at earlier stages, by preventing the activation and release of TGFβ from LAP (Latency Associated Peptide) using specific antibodies to effectively prevented the integrin-mediated activation of latent TGFβ (37, 38) and was, along with other TGFβ blockers (1D11 and GC1008) tested in clinical trials (10) or more conventional approaches such as immune checkpoint inhibitors. Systemic antibody infusions may cause side effects, acute reactions such as anaphylactic responses, cardiotoxic effects, dermatitis or autoimmune reactions (39). Cytokine Release Syndrome (CRS), a potentially fatal adverse effect, may also occur following antibody-based therapy infusion (40). In contrast, we have shown herein that trap secretion is dependent on the activation level (**Figure 1H**). Additionally, the retroviral platform we have used has been demonstrated to display reduced expression overtime, but upon T cell (re)activation, transgene production is upregulated again (41). This would contribute to limit trap production to the immediate surroundings of antigen expressing cells, understandably reducing potential adverse effects.

Antibodies can be engineered in various forms, ranging from their full form with modifications to small single-chains or nanobodies (42). Despite their small size, single-chain anti-TGFβ traps can function as effectively as full anti-TGFβ antibodies (43). Bispecific antibodies can also be harnessed in the context on engineered T cells as it was recently shown in a study in which CD19 CAR T cells were modified to express an anti-PD1/TGFβ molecule, although this work focused on a hematological cancer model lacking solid tumor TME and did not assess the contribution of the TGFβ blocking moiety alone (44). Nonetheless, potent *in vivo* activity was observed, lending support to the present approach.

As expected, we did not observe significant improvements in the expression of different activation markers in trap-expressing T cells which lack the co-stimulatory component compared to CSRs. However, the *in vivo* assay we performed in this study revealed a superior activity by trap-expressing T cells, suggesting the importance of widely targeting and blocking TGFβ in the TME rather than only providing co-stimulation to selected T cells. We thus assume this approach may show promise for adoptive cell therapy, whether using receptor engineered T, NK cells or naturally occurring tumor infiltrating lymphocytes and other immune cells (45). The present strategies could also be combined with T-cells metabolically engineered to cope with the lack of nutrients in the TME (46, 47) future research should explore the combination of our approaches with other immunotherapy strategies, such as checkpoint inhibitors or cancer vaccines, to potentially achieve synergistic effects (15).

Our study, while promising, has limitations that could be acknowledged. First, the potential for off-target effects of both the chimeric switch receptors (CSRs) and the TGFβ trap will require further exploration. While we observed improved T cell function, there is a possibility that altering TGFβ signaling could affect other cellular processes or non-cancerous tissues, given TGFβ’s diverse roles in the body. Second, our findings are primarily based on a melanoma model, and their applicability to other cancer types remains to be established. While TGFβ secretion by cancer cells and resident immune cells has been established for tumors of various histologies, different tumor microenvironments may respond differently to these approaches. Finally, the scalability of producing engineered T cells expressing CSRs or TGFβ traps for clinical use may present challenges. Nonetheless, it was shown it was possible to incorporate the use of PD1 CSR engineered T-cells in clinical trial settings (21) which strengthens the feasibility of this strategy. In parallel, it will be interesting to corroborate our findings in an immunocompetent mouse model encompassing all the components of an active immune system. While we recognize the ethical complexities of using animal models in cancer research, these are often necessary to better characterize the therapeutic potential of immune interventions in pre-clinical settings (48).

In conclusion, we demonstrated that TGFβRI-based CSR and TGFβ trap can improve TCR T cells function. Ultimately, our work contributes to the growing toolkit of cancer immunotherapy and underscores the importance of targeting the tumor microenvironment to enhance anti-tumor immune responses. We trust these approaches will hold promise for the betterment of cellular immunotherapy of cancer.

## Data Availability

The original contributions presented in the study are included in the article/Supplementary Material. Further inquiries can be directed to the corresponding author/s.
